# Assessment of Albumin Usage Patterns and Appropriateness in a Comprehensive Cancer Centre: A retrospective study in Jordan

**DOI:** 10.18295/squmj.8.2024.049

**Published:** 2024-08-29

**Authors:** Asma’a A. Al-Kharabsheh, Lama H. Nazer, Wedad Awad, Ala'a Ghanem, Rand Al-Hadaddin, Batool Bani Amer, Hadeel Thawaibeh, Nour Mustafa, Rula Al-Najjar, Abeer Al-Rabayah, Saad Jaddoua

**Affiliations:** Department of Pharmacy, King Hussein Cancer Center, Amman, Jordan

**Keywords:** Albumin, Therapeutic Use, Drug Utilisation Review, Neoplasms, Cancer Care Facilities, Jordan

## Abstract

**Objectives:**

Albumin is commonly used for various indications; however, there is conflicting data regarding its appropriate use in different clinical cases. This study aimed to determine the pattern and appropriateness of albumin use among cancer patients at the King Hussein Cancer Center in Jordan.

**Methods:**

A retrospective analysis was conducted on adult cancer patients who were prescribed albumin between January 2019 and July 2020 in both outpatient and inpatient settings. Data collected included demographics, prescribing services, indications and dosing regimens. A literature review was performed using PubMed to assess the appropriateness of albumin indications and dosing regimens against current guidelines, drug information resources and the package insert.

**Results:**

Albumin was prescribed to 1,361 patients during the study period. Each patient received an average of 74.4 ± 89 g of albumin for an average of 2.6 ± 1.8 days. Albumin use was deemed appropriate in 69% of the patients. The critical care service accounted for the highest albumin consumption, with 37% of prescriptions for septic shock. Inappropriate use of albumin was most prevalent in the medical solid tumour services (40.8% of prescriptions), primarily for edema (28%).

**Conclusion:**

To the best of the author’s knowledge, this study is the first to evaluate albumin use in a large cohort of oncology patients. Approximately one-third of the albumin prescriptions were considered inappropriate. Continuous education on appropriate usage and regular evaluations of guideline adherence are essential to ensure proper utilisation of albumin in cancer care.

**Advances in Knowledge**

- Over one-third of the studied adult patients received albumin for purposes that did not align with international guidelines of cancer-related albumin use.

**Applications to Patient Care**

*- Considering albumin’s scarcity and cost, this study asserts the need to establish guidelines on cancer-related albumin use supported by consensus and evidence*.
*- Additionally, it foregrounds the necessity of ongoing education regarding proper albumin usage and consistent evaluations of albumin use to ensure the successful implementation of these guidelines*.

Human albumin, a physiological plasma expander, is commonly used for various indications such as septic shock, paracentesis-induced circulatory dysfunction, spontaneous bacterial peritonitis and hepatorenal failure.^1^–^3^ However, there are conflicting literature data regarding the clinical effectiveness of albumin in various clinical scenarios. An example of this conflict is the Saline versus Albumin Fluid Evaluation (SAFE) trial, which demonstrated that 4% albumin resulted in outcomes comparable to those of normal saline when used for fluid resuscitation over a 28-day period.^4^ Additionally, a Cochrane review concluded that there was insufficient evidence to establish the superiority of colloids over crystalloids in reducing mortality in fluid resuscitation.^5^

Recent studies have examined potential additional benefits of albumin, including its anti-inflammatory and antioxidant properties, binding capacity, modulation of haemostasis, vasodilatation and acid-base homoeostasis.^6^^,^^7^ These studies have led to an increase in its global prescription, with indications often extending to unapproved uses lacking robust clinical evidence.^8^^,^^9^ The heterogeneity of patients’ baseline characteristics and albumin levels has also contributed to the limited availability of clinical evidence regarding the benefits of albumin use.

Existing literature describes the use of albumin among cancer patients with various clinical conditions. It is well-documented that cancer patients admitted to intensive care units (ICUs) frequently exhibit hypoalbuminemia, defined by serum albumin levels falling below 2 g/dL.^10^ Furthermore, albumin is commonly prescribed for paracentesis procedures following the occurrence of ascites, irrespective of whether the underlying cause is malignancy-related.^11^ Despite these advances, a comprehensive assessment of the appropriateness of albumin usage and its indications is absent from the existing literature. Notably, the only study available for such an evaluation involves only 53 patients.^13^

This study aimed to bridge this literature gap by examining the appropriateness of cancer-related albumin utilisation in a comprehensive cancer centre, including an assessment of the indications and dosing regimens.

## Methods

This study, conducted at the King Hussein Cancer Centre (KHCC) in Amman, Jordan, was retrospective in nature. KHCC is a 350-bed comprehensive cancer centre that treats adult and paediatric patients with various types of malignancies in both inpatient and outpatient settings.

Utilising KHCC’s pharmacy electronic system, the study identified all patients prescribed albumin between January 2019 and July 2020 at KHCC. The study included adult patients (≥18 years of age), with a history of cancer who received albumin in inpatient or outpatient settings. Patients who received albumin as a fluid expander for stem cell collection (for the extracorporeal photopheresis procedure) were excluded from further assessment.

Each instance of albumin prescription during the study period was evaluated for the appropriateness of both the prescribing indication and the dosage regimen. The product available at KHCC contained 20% albumin in 50 ml. To determine the appropriateness of albumin indications and dosing regimens, a team of 3 clinical pharmacists thoroughly reviewed the literature and related guidelines. This team subsequently formulated a list of evidence-based albumin indications and dosing regimens based on the most relevant literature, outlined in Supplementary Table 1.^1^^,^^3^^,^^4^^,^^14^ This list was used by the reviewers to assess the appropriateness of albumin use among the study patients.

This study utilised the American Society of Health-System Pharmacists (ASHP) guidelines for medication use evaluation to conduct its Drug Use Evaluation (DUE).^12^ Albumin use indications were determined based on the indications included in physicians’ notes on patients’ electronic medical records. In cases where the indication for albumin prescription was unclear, a second pharmacist reviewed each case. If there was any disagreement between the two reviewers, a third reviewer examined the patient’s profile.

Patient characteristics, including age, weight, gender, type of malignancy and albumin serum levels, were recorded using the electronic patient medical record. Additionally, the study determined the albumin dose, indication, treatment duration and number of albumin vials dispensed. To identify the clinical services most frequently prescribing albumin, the study reviewed electronic prescriptions of albumin.

The evaluation of medication cost per service was determined using the electronic billing system of KHCC, with the associated expense based on the institutional selling price. The quantity of vials was calculated considering the availability of 50 ml vials of 20% albumin, rounding to a full vial.

Data analysis was conducted using Excel, Version 2016 (Microsoft Inc., Redmond, Washington, USA). Descriptive statistics were used: average ± standard deviation for continuous data and numbers and percentages for nominal data. The study was approved by the KHCC institutional review board and was granted a waiver of informed consent, owing to its retrospective nature (21 KHCC 002).

## Results

During the study period, 1,361 patients were included and a total of 2,399 albumin prescriptions were identified. Among the patients, over half were male, the average patient age was 60 ± 16 years. The majority received albumin in the inpatient setting (83.6%). Each patient received an average of 74.4 ± 89 g of albumin, equivalent to 7.44 ± 8.9 vials. The average duration of albumin use was 2.6 ± 1.8 days [Table 1].

**Figure 1:**
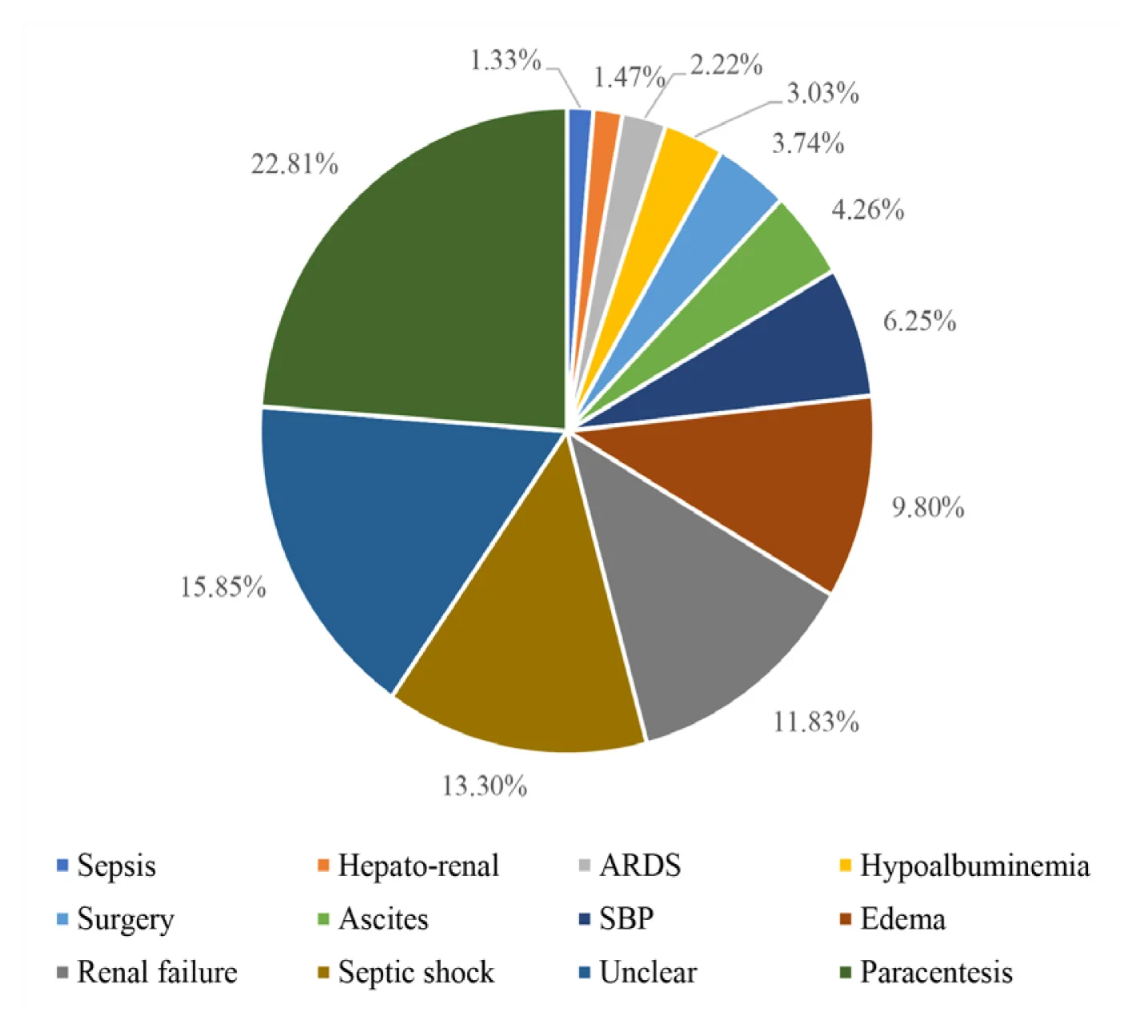
Distribution of albumin prescriptions according to the indications (%) *ARDS = acute respiratory distress syndrome; SBP = spontaneous bacterial peritonitis

The most frequent indications for albumin prescription were paracentesis (22.8%), septic shock (13.3%) and renal failure (11.8%) [Figure 1]. Regardless of the appropriateness, the clinical service with the highest number of albumin prescriptions was the medical solid tumour service (n = 733; 29.5%), followed by the nephrology service (n = 537; 21.6%) and the critical care service (n = 352; 18.2%) [Table 2].

Most albumin consumption was attributed to hospitalised patients (85.8%), while outpatient settings constituted a smaller proportion (14.2%). In outpatients, the most common reason for albumin use was fluid replacement following paracentesis (n = 96; 27 %). For each such procedure, an average of 30 g dose of albumin was administered once post-procedure.

Of the patients included in the study, 69% were prescribed albumin for indications aligned with current clinical evidence and internationally available guidelines.^1^^,^^14^ As summarised in Table 2, the solid tumour service had the highest percentage of inappropriate albumin prescriptions relative to its total number of prescriptions, with 299 cases (40.8%). This was also true for the surgical service, with 128 cases (65.6%), followed by the palliative service. Among patients who received albumin for appropriate indications, incorrect dosing regimens were reported in 385 (23.1%) prescriptions.

The total quantity of albumin used during the study period was 183,290 g, equivalent to 18,329 vials. Of this, 136,070 g of albumin were prescribed appropriately. The total cost of albumin consumption amounted to 678,173 Jordanian Dinar (JD; equivalent to 955,173 USD). Notably, the expenditure of albumin for inappropriate indications and/or dosing regimens was 174,714 JD (equivalent to 246,076 USD).

## Discussion

This study, with its relatively large sample size, reported inappropriate albumin prescriptions in approximately one-third of the cases. This proportion is higher than that reported by other studies, which could be attributed to several factors such as the presence of clinical pharmacists in the inpatient setting across all clinical services to assess the necessity of albumin use and the implementation of a strict duration requirement for the prescription of this medication.^15^–^17^

The inappropriate utilisation of albumin has been well-documented in various studies globally.^8^^,^^9^^,^^15^–^17^ These findings highlight the importance of establishing institutional consensus guidelines to regulate albumin use, minimise medication wastage and prevent unintended adverse drug reactions.

Jahangard-Rafsanjani *et al*. evaluated albumin use in a teaching hospital in Iran, including 135 patients and reported appropriate use in only 34% of the cases.^15^ Similarly, Nafisi *et al*. conducted a study in Urmia, Iran, between 2014 and 2015, involving 202 patients, and found a 39.1% rate of appropriate albumin prescription.^16^

In Tehran, Iran, Talasaz *et al*. identified hypoalbuminemia and nutritional supplementation as primary inappropriate indications for albumin use, accounting for 36.3% and 24.4% of inappropriate uses, respectively.^17^ Nafisi *et al*. also reported similar findings, with 13.4% of albumin prescriptions falling into these categories.^16^

This study also observed significant instances of nutritional support and hypoalbuminemia as inappropriate indications for albumin use, accounting for 10% (n = 89) of all inappropriate indications. Oncology patients often experience malnutrition and cachexia due to their disease and its treatment, which likely contributes to these inappropriate prescriptions.^18^

Though albumin is not recommended as a caloric protein source nor to maintain serum albumin levels above 3 g/dL, prescribing albumin to increase serum levels remains a common practice. Hypoalbuminemia, in many cases, can be attributed to the impact of inflammatory processes on serum albumin levels, which leads to increased vascular permeability and leakage of albumin molecules.^19^–^21^ However, based on current evidence, albumin infusions cannot be recommended solely to increase serum albumin levels. Instead, it is crucial to identify and treat the underlying cause of hypoalbuminemia.^18^

In the current study, the ICU accounted for the highest consumption of albumin vials, receiving 37% of the total number of albumin vials dispensed in the study period. In the ICU, albumin was commonly prescribed as a volume expander for conditions such as septic shock. Notably, the use of albumin in cases of septic shock, as highlighted by Tigabu *et al*., did not demonstrate a reduction in the 28-day mortality rate and was not considered cost-effective for this indication.^22^^,^^23^ However, albumin could have a beneficial effect on patients receiving large volumes of crystalloids, as these patients presented with higher blood pressure at early and later time points, higher static filling pressures and lower net fluid balance.^1^

Paracentesis emerged as a common indication for appropriate albumin use. However, it is important to note the lack of uniformity among globally recognised guidelines concerning the exact volume of fluid extraction necessitating albumin administration.^3^ This study’s analysis revealed that a significant portion of the selected patients’ medical records lacked detailed information regarding the precise quantity of fluid withdrawn during paracentesis. Consequently, all patients receiving albumin for paracentesis were deemed appropriately treated. Nonetheless, this aspect highlighted a limitation of this study—it was unable to ascertain whether the drained fluid volume aligned with the prescribed albumin dose for replenishment and the suitability of such a prescription.

Patients with chronic renal disease typically exhibit low levels of serum albumin, primarily due to reduced albumin synthesis. This reduced synthesis can be caused by malnutrition resulting from both anorexia related to uraemia and protein restriction imposed during the advanced stages of renal insufficiency. Additionally, patients with nephrotic syndrome may experience significant albumin loss in the urine.^24^ In this study, the nephrology service accounted for approximately 21% of albumin prescriptions. Among these prescriptions, only 5.8% were reportedly used inappropriately, mostly to treat edema, especially in cases of diuretic resistance. According to Lee *et al*., the utilisation of albumin in cases of diuretic resistance lacks a solid foundation of evidence due to the varied nature of relevant data and the absence of clearly proven benefits concerning the enhancement of diuresis.^25^ These observations underscore the need for additional education to address this issue and mitigate inappropriate albumin usage.^25^

In this respect, Buckley *et al*. reported a significant reduction (50.9%) in inappropriate albumin prescribing after implementing pharmacist-led interventions and medication prescribing reviews.^26^ Their findings highlight the crucial need for developing guidelines and establishing strict criteria to control the prescribing patterns of albumin in hospitals and prevent resource waste. Considering the findings of this study, the authors aim to subsequently develop local consensus guidelines regarding albumin use in the KHCC and conduct additional educational sessions to further enhance and standardise albumin utilisation.

The results of this study, along with other studies documented in the literature, collectively indicate a consensus regarding the inappropriate use of albumin. Going forward, DUEs will play a significant role in fostering discussions between physicians and pharmacists to obtain and develop evidence-based guidelines for the appropriate use and optimal utilisation of albumin.

However, this study has some other limitations that should be acknowledged. First, its retrospective design and reliance on patient medical records and physician notes as the sole source of information might have introduced potential biases and led to incomplete documentation. Its retrospective nature also limited the authors’ ability to evaluate the clinical outcomes or complications associated with the use of albumin. Second, the presence of multiple reported indications involving albumin use posed a challenge to determining the appropriateness of these indications and identifying the primary indication for use. To mitigate this limitation, patients with unclear indications were subjected to second and third reviews of their clinical notes and medication profiles; through them, the authors attempted to enhance the accuracy of determining the most likely primary indications and their appropriateness. Another limitation of this study was the absence of a comprehensive pharmacoeconomic analysis regarding the use of albumin; its analysis did not encompass the effect of albumin on patient outcomes (e.g., length of stay, mortality), the indirect costs associated with the preparation and administration of albumin or the potentially adverse events associated with its use. This study’s analysis was confined to the direct costs of albumin use.

## Conclusion

This study reveals the inappropriate use of albumin in approximately one-third of the relevant cancer cases at a comprehensive cancer centre in Jordan. Given albumin’s limited availability and cost, the development of evidence-based guidelines addressing the primary indications for albumin use is recommended. Additionally, continuous education on proper albumin usage and regular evaluations of guideline implementation should be considered. Future research should focus on evaluating the impact of interventions aimed at promoting evidence-based albumin prescription and assessing the effects of albumin use on patient outcomes, such as 28-day mortality and ICU length of stay, as well as adverse events.

## Supplementary Information



## Figures and Tables

**Table 1:** Baseline characteristics of the patients included in the study (N = 1,361)

Characteristic	n (%)
**Gender**	
Male	743 (54.6)
Female	618 (45.6)
**Patient status at the time of albumin order**	
Inpatient	2,078 (83.6)
Outpatient	407 (16.4)
**Age in years, mean ± SD**	60 ± 16
**Type of malignancy**	
Breast cancer	155 (11.3%)
Lung cancer	117 (8.5%)
Colorectal cancer	84 (6.1%)
Gynaecological malignancies	112 (8.3%)
Pancreas cancer	53 (3.9%)
Others	840 (61.7%)
Serum albumin at the time of prescription in g/dL, mean ± SD	2.62 ± 0.6
Number of vials per patient, mean ± SD	7.44 ± 8.9
**Duration of albumin use in days, mean ± SD**	2.6± 1.8
**Total number of albumin vials dispensed**	18,329
**Total cost of used albumin vials in USD**	960,471

**Table 2:** Albumin prescriptions across various services and the number of prescriptions considered inappropriate within each service (N = 1,361)

Service*	n (%)	
	Albumin prescriptions per service	Inappropriate prescriptions†
Solid tumours	733 (29.5)	299 (40.8)
Nephrology	537 (21.6)	31 (5.8)
Critical care	452 (18.2)	58 (12.8)
Outpatient	354 (14.2)	189 (53.3)
Palliative	134 (5.4)	58 (43.2)
Surgical	128 (5.2)	84 (65.6)
Lymphoma	38 (1.5)	13 (34.2)
Leukaemia	23 (0.9)	9 (39.1)

* Refers to the clinical service that prescribed albumin for the patient, regardless of the patient’s type of malignancy or hospital location.

† The percentage of inappropriate prescriptions compared to the total number of prescriptions within the same service.
